# Esmolol’s Role in Hemodynamic Management During Pheochromocytoma Surgery: A Comprehensive Review

**DOI:** 10.7759/cureus.61786

**Published:** 2024-06-06

**Authors:** Pavithra Konjety, Vivek Chakole

**Affiliations:** 1 Anaesthesiology, Jawaharlal Nehru Medical College, Datta Meghe Institute of Higher Education and Research, Wardha, IND

**Keywords:** perioperative care, catecholamines, surgery, hemodynamic management, pheochromocytoma, esmolol

## Abstract

Pheochromocytoma (PCC) surgery presents significant challenges due to the hemodynamic instability induced by catecholamine release. Effective perioperative management is essential to minimize complications and ensure optimal outcomes. This comprehensive review examines the role of esmolol, a short-acting beta-blocker, in hemodynamic stabilization during PCC surgery. We provide an overview of the pathophysiology of PCC, highlighting the cardiovascular effects of excessive catecholamines. Challenges in perioperative management and the need for effective hemodynamic control are discussed. The pharmacology and mechanisms of action of esmolol are outlined, along with evidence from clinical studies supporting its use in PCC surgery. Comparative analyses with other hemodynamic agents are presented, along with recommendations for optimizing esmolol administration and monitoring. Key findings include the ability of esmolol to attenuate catecholamine-induced hypertension and tachycardia, thereby promoting hemodynamic stability and reducing the risk of intraoperative cardiovascular crises. Implications for clinical practice include the incorporation of esmolol into perioperative management protocols and the importance of multidisciplinary collaboration. Future research directions include further elucidating optimal dosing regimens, comparative effectiveness studies, and exploring novel therapeutic approaches. Collaboration among clinicians, researchers, and pharmaceutical companies is essential to advance the care of patients undergoing surgery for PCC.

## Introduction and background

Pheochromocytoma (PCC), a rare neuroendocrine tumor originating from chromaffin cells in the adrenal medulla or extra-adrenal paraganglia, represents a challenging clinical entity. These tumors exhibit the aberrant secretion of catecholamines, notably epinephrine and norepinephrine, resulting in intermittent or sustained hypertension and various cardiovascular manifestations [1]. While surgical resection is the cornerstone of treatment for PCC, the procedure presents significant hurdles due to the hemodynamic instability induced by catecholamine release during tumor manipulation. These challenges encompass profound hypertension, tachycardia, arrhythmias, and potential intraoperative cardiovascular crises [2].

Effective hemodynamic management is paramount to achieving favorable surgical outcomes and mitigating perioperative complications in patients undergoing PCC surgery. Inadequate control of blood pressure and heart rate can precipitate life-threatening cardiovascular events, including myocardial infarction, stroke, or pulmonary edema [3]. Therefore, this comprehensive review explores the role of esmolol, a short-acting beta-blocker, in hemodynamic management during PCC surgery. By examining esmolol's pharmacology, mechanisms of action, clinical evidence, and comparative efficacy against other hemodynamic agents, this review offers valuable insights into optimizing perioperative care for individuals afflicted with PCC.

## Review

Standard hemodynamic management techniques

Preoperative Preparation

The preoperative preparation for patients diagnosed with PCC necessitates a comprehensive strategy to optimize blood pressure, intravascular volume, and hormonal balance to minimize the risk of perioperative complications. Alpha-adrenergic antagonists represent the recommended initial pharmacological choice for premedication to achieve optimal alpha-blockade before surgery [3]. Beta-adrenergic antagonists are used as adjunctive therapy alongside alpha-blockers to regulate heart rate and prevent tachycardia [3]. Calcium channel blockers may also be incorporated into the pharmacological regimen to help regulate blood pressure and heart rate [3]. The medication regimen can include tyrosine hydroxylase inhibitors to address excessive catecholamine production [3]. Fluid therapy is critical in restoring blood volume, typically utilizing saline solution and encouraging salt consumption, particularly in patients exhibiting chronic vasoconstriction [3]. Insulin therapy is employed as necessary to manage hyperglycemia as part of the preoperative optimization process [3]. The overarching goal of preoperative preparation is to attain a seated blood pressure target of <130/80 mm Hg while avoiding standing systolic blood pressure <90 mm Hg and maintaining heart rate within recommended values. This meticulous preparatory approach significantly mitigates the morbidity and mortality rates associated with PCC surgery. The optimization of blood pressure, intravascular volume, and hormonal balance is paramount in preventing hypertensive crises and other adverse hemodynamic events during and following surgery [4].

Intraoperative Management

Precision and careful execution are indispensable to maintain stable hemodynamics in the presence of catecholamine surges during critical junctures like laryngoscopy, peritoneal insufflation, surgical stimulation, and tumor manipulation [5]. Effective coordination between the surgical and anesthesia teams is paramount to navigate these surges and address the subsequent potential of hypotension following tumor ligation [5]. Anesthetic strategies capable of adeptly managing catecholamine surges are imperative, with sevoflurane emerging as the predominant inhalational agent for maintaining anesthesia [6]. In patients with cardiomyopathy, propofol or etomidate are favored for induction, while ketamine is advised against due to its sympathomimetic properties [6]. Ensuring a smooth induction of anesthesia at an optimal depth is crucial to mitigate hypertensive responses during laryngoscopy and intubation, which may be amplified in individuals with PCC [6]. Utilization of invasive hemodynamic monitoring, including arterial lines and central venous access, is advocated to facilitate prompt adjustment of vasopressors and inotropes as warranted [6]. In cases of severe cardiac dysfunction, consideration may be given to employing transesophageal echocardiography (TEE) or pulmonary artery catheters [6]. Early ligation of the adrenal vein is recommended to diminish the probability of significant catecholamine release [7].

Postoperative Care

Patients receive meticulous monitoring in the intensive care unit to detect hemodynamic fluctuations, particularly instances of hypotension and hypertension [8]. Brief episodes of hypotension are frequently encountered, attributable to residual alpha-blockade, preoperative volume depletion, and intraoperative blood loss. Management typically involves intravenous hydration and/or administration of vasopressors [7]. Any drowsy or unresponsive patients necessitate evaluation for electrolyte and endocrine abnormalities, with hypoglycemia and hyponatremia ranking high on the list of potential diagnoses. Vigilant monitoring of blood glucose and electrolyte levels is imperative. Fluid management is carefully regulated, given the postoperative risk of hypotension and hypoglycemia [8]. Persistently hypertensive patients prompt consideration of fluid overload, restoration of autonomic reflexes, unintended ligation of the renal artery, or residual tumor presence [8]. Potential complications include fever, hypoglycemia, cortisol deficiency, and urinary retention [8]. Accurate diagnosis and prompt treatment of these emergent situations are paramount [8]. Postoperative care aims to ensure timely intervention in cases of tumor recurrence, incomplete resection, or metastasis development. Long-term annual fractionated metanephrine testing has been recommended, even in cases where postoperative levels appear normal [8].

Role of esmolol in hemodynamic stabilization

Pharmacology of Esmolol

**Figure 1:**
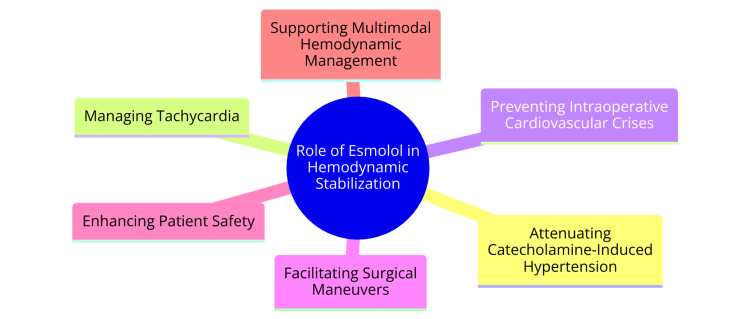
Role of esmolol in hemodynamic stabilization Image Credit: Dr Pavithra Konjety

Esmolol, an ultra-short-acting beta-adrenergic blocking agent, is distinguished by its minimal partial agonist activity and absence of direct membrane depressant effects [9]. Its cardioselective nature renders it an invaluable tool in managing diverse cardiovascular conditions. Esmolol functions through competitive antagonism of the β-1-adrenergic receptor, resulting in reduced heart rate and contractility, thus alleviating cardiac workload and oxygen demand [10]. This selective β-1 blockade proves, particularly, efficacious in addressing supraventricular tachycardias like atrial fibrillation, atrial flutter, and hypertension induced by intubation [10]. The brief duration of esmolol's action stems from its rapid enzymatic hydrolysis by red blood cell esterases, yielding ASL-8123 and methanol [9]. This swift metabolism enables rapid titration to attain the desired steady-state level of beta-blockade, facilitates prompt adjustment to different levels of the beta-blockade as necessary, and ensures rapid beta-blockade dissipation upon discontinuing esmolol infusion [9]. Indicated for short-term use, esmolol is effective in managing rapid ventricular rates in individuals with atrial fibrillation or atrial flutter and intubation-induced hypertension [10]. Its off-label applications include managing rate and rhythm in conditions such as aortic dissection, acute coronary syndrome, non-ST elevation myocardial infarction, hypertensive emergencies, thyrotoxicosis, refractory ventricular tachycardia, ventricular fibrillation resistant to defibrillation, and attenuating the catecholamine response during electroconvulsive therapy [10]. Demonstrated to be both safe and efficacious, esmolol is particularly advantageous for blood pressure control during surgical procedures owing to its abbreviated half-life [10]. Its utility extends across clinical scenarios, encompassing urgent care, perioperative management, and postoperative care [10]. The role of esmolol in hemodynamic stabilization is shown in Figure 1.

Mechanisms of Action in PCC Surgery

Controlling catecholamine release is paramount in managing PCCs, as these tumors secrete excessive amounts of catecholamines such as epinephrine, norepinephrine, and dopamine, which can precipitate hypertensive crises during tumor manipulation. Preoperative alpha-adrenergic blockade, typically achieved with medications like phenoxybenzamine or doxazosin, is crucial in averting such crises [8,11,12]. Ensuring hemodynamic stability throughout the pre- and intra-operative periods is essential. Esmolol, a short-acting beta-blocker, controls paroxysmal hypertension and tachycardia during these critical phases. By reducing heart rate and cardiac output, esmolol helps maintain hemodynamic equilibrium [8,11,12]. Following the removal of the PCC, there is a rapid decline in catecholamine levels, often resulting in postoperative hypotension. To counteract this, aggressive fluid resuscitation and vasopressor support are indispensable [3,8]. PCCs can induce metabolic derangements, including hyperglycemia by inhibiting insulin secretion. Conversely, postoperatively, there is a risk of hypoglycemia as catecholamine levels plummet. Close monitoring and appropriate intervention are essential to manage these metabolic fluctuations effectively [3,8].

Clinical Studies Evaluating Esmolol Use

Clinical studies investigating the utilization of esmolol have consistently demonstrated its effectiveness and safety across various medical contexts. In patients with supraventricular tachycardia and those requiring intraoperative and postoperative care, esmolol has proven to be well-tolerated, with the majority of adverse effects being mild and temporary, the most prevalent being hypotension [13]. Moreover, early administration of esmolol in septic shock has been linked to favorable hemodynamic effects, including heightened stroke volume and diminished norepinephrine requirements, without adverse impacts on organ function [14]. Esmolol's application in hyperkinetic septic shock has been associated with a reduced cardiac index primarily due to its negative chronotropic effect, underscoring its significance in optimizing hemodynamics [14]. Additionally, the short half-life of esmolol facilitates rapid reversal of potential adverse hemodynamic effects, rendering it a valuable option across diverse clinical scenarios, encompassing surgical and urgent care settings [10]. Collectively, clinical studies have underscored esmolol's efficacy in heart rate control, improvement of hemodynamics, and enhancement of patient outcomes in various medical conditions.

Benefits and Limitations

Esmolol is effective in managing rapid heartbeats or irregular heart rhythms and addressing elevated heart rate and blood pressure during surgical procedures, postoperative recovery, or other medical interventions [15]. In patients with septic shock, esmolol therapy aimed at reducing heart rate has demonstrated positive impacts on hemodynamics, including increased stroke volume, maintenance of mean arterial pressure, and decreased requirements for norepinephrine, all without adverse effects on organ function [15]. Notably, esmolol administration significantly reduced heart rate at various time points, including 12, 24, 48, and 72 hours, among adults with sepsis and septic shock [16]. Moreover, there is growing evidence suggesting that esmolol treatment may contribute to decreased 28-day mortality, heart rate control, and cardioprotective effects in patients with sepsis or septic shock following early fluid resuscitation [16]. Additionally, esmolol has demonstrated the ability to significantly lower serum troponin I concentrations, a marker indicative of myocardial dysfunction, thus potentially predicting heightened sepsis severity and increased mortality rates [16].

However, it is important to note that esmolol is not recommended for use in pediatric patients below 18 years of age due to the lack of established safety and efficacy data [17]. Moreover, existing evidence does not provide conclusive information regarding whether esmolol affects the length of ICU stay or the PaO_2_/FiO_2_ ratio in patients with sepsis and septic shock, as the quality of evidence was deemed "very low" [16]. Variations in individual myocardial inhibition among sepsis patients and differences in esmolol treatment methods may have influenced the pooled results, emphasizing the need for further investigation into the optimal dosing regimens and administration protocols for esmolol in this population [16]. Larger randomized controlled trials are warranted to validate these findings and assess for potential publication bias [16].

Comparative analysis with other hemodynamic agents

Beta-Blockers versus Alpha-Blockers

It is crucial to grasp their mechanisms of action and clinical applications when comparing beta-blockers and alpha-blockers. Beta-blockers, exemplified by esmolol, function by obstructing beta-adrenergic receptors, thereby diminishing the sympathetic effects on the cardiovascular system. They find widespread use in treating a spectrum of conditions, including hypertension, heart failure, myocardial infarction, angina, and arrhythmias [18]. Conversely, alpha-blockers like phentolamine and phenoxybenzamine impede the binding of norepinephrine to smooth muscle receptors, resulting in vasodilation and consequent blood pressure reduction. They prove particularly efficacious in managing hypertension stemming from conditions such as PCC [19]. Considering side effects, beta-blockers may induce common manifestations like fatigue, cold extremities, and dizziness, while rarer occurrences encompass depression and memory impairment. Conversely, alpha-blockers can prompt tachycardia and cardiac arrhythmias due to their non-selective nature and impact on alpha-2 receptors [19]. While both categories of blockers possess distinct applications and side effect profiles, the selection between beta-blockers and alpha-blockers hinges on the specific condition under treatment and the individual patient's requirements. Beta-blockers are predominantly employed for cardiovascular ailments, whereas alpha-blockers are favored for conditions necessitating vasodilation and blood pressure reduction, as observed in managing PCC-induced hypertension [20,21].

Efficacy and Safety Profiles

Extensive research has shed light on esmolol's efficacy and safety profiles across various medical contexts. Findings suggest that when titrated to a hemodynamic endpoint, esmolol emerges as a safe and effective option, particularly in low-risk patients, underscoring its potential to modulate hemodynamic responses during medical procedures [22]. Investigations into esmolol dose titration have revealed its influence on changes in arterial blood pressure and heart rate, with notable findings indicating a significant reduction in the frequency of myocardial ischemia compared to placebo [23]. Furthermore, studies focusing on the safety and efficacy of esmolol in noncardiac surgery settings have underscored its role in mitigating perioperative myocardial infarction while preserving hemodynamic stability [23]. The distinctive attributes of esmolol, including its selective beta-1 antagonism, rapid onset of action, and short duration, render it a favorable choice for managing hemodynamic responses. However, caution is warranted when administering esmolol in conjunction with specific calcium channel blockers due to the potential risk of hypotension and AV-conduction disorders [24].

Clinical Guidelines and Recommendations

Esmolol distinguished as a selective beta-blocker, offers effectiveness, titratability, and rapid weaning capabilities, rendering it an optimal choice for managing labile hypertension in children afflicted with PCC. This is particularly advantageous when conventional beta-blockers are deemed unsuitable due to concurrent conditions such as asthma [5,25]. In concert with alpha-blockade, esmolol proves instrumental in effectively controlling hypertension among pediatric patients with PCC, thereby mitigating perioperative complications [25]. Its recognized safety and efficacy in blood pressure regulation during surgical procedures, attributed to its short half-life, confer significant value across clinical settings, spanning urgent care, perioperative management, and postoperative recovery [10]. Esmolol's pivotal role in managing intraoperative hypertensive crises and attenuating postoperative hypotension is instrumental in ensuring favorable outcomes for individuals undergoing surgery for PCC [26].

Optimizing esmolol administration

Dosage Regimens

Esmolol Hydrochloride 10 mg/mL solution for injection typically utilizes an effective dosage ranging from 50 to 200 mcg/kg/min, although in certain cases, doses as high as 300 mcg/kg/min may be warranted [27]. In the event of adverse reactions such as bradycardia, bronchospasm, symptomatic hypotension, or cardiovascular depression, specific interventions like atropine, beta-2-sympathomimetics, fluid resuscitation, pressor agents, or inotropic agents may be required [27]. A novel dosing regimen for esmolol infusion has been devised to promptly manage supraventricular tachyarrhythmia post-cardiac surgery, initiating with an infusion rate of 150 or 100 mcg/kg/min, adjusted based on the patient's age and blood pressure [28]. This updated regimen has demonstrated safety and efficacy in controlling tachycardia among Chinese patients, maintaining an infusion rate averaging 73 +/- 42 mcg/kg/min [28]. In pediatric intensive care settings, esmolol dosing optimization is facilitated through computerized practitioner order entry (CPOE), commencing with an initial dose of 50 mcg/kg administered over five to 10 min, followed by infusion initiation at 100 mcg/kg/min, with subsequent titration every 15 min, up to a maximum dose of 300 mcg/kg/min [29]. This study aims to delineate esmolol's hemodynamic/cardiovascular dose response in pediatric care, emphasizing the criticality of vigilant monitoring and dosage adjustment to ensure both efficacy and safety [29]. For conditions such as atrial fibrillation, atrial flutter, supraventricular tachycardia, or intra- or post-operative supraventricular tachycardia or hypertension, esmolol is typically administered via continuous intravenous infusion, optionally with a loading dose, with maintenance infusion rates tailored to achieve the desired therapeutic response [30]. This dosing regimen may encompass optional loading doses followed by maintenance infusion rates ranging from 50 to 150 mcg/kg/min, contingent upon the patient's individual response to treatment [30].

Monitoring Parameters

Vigilant monitoring of parameters during esmolol administration is paramount to uphold patient safety and efficacy. Close scrutiny of cardiovascular metrics such as blood pressure and heart rate is imperative throughout the process [27]. Recommended monitoring protocols entail an initial assessment of baseline vital signs and ECG rhythm before initiation, followed by continuous monitoring every five minutes post-rate adjustment until the desired heart rate and blood pressure levels are attained. Subsequently, monitoring frequency may transition to every five hours [31]. Additionally, meticulous attention is warranted to ascertain the concentration and validate the dosage with each bag change, conducted within one hour of assuming patient care or sooner if clinically indicated. This practice ensures precise dosing alignment with each rate adjustment [32]. Continuous surveillance for adverse reactions and potential side effects, including early signs of impending congestive heart failure or bronchoconstriction, remains imperative to promptly address any emergent complications associated with esmolol infusion [32]. Given the propensity for hypotension, particularly with infusion rates surpassing 200 mcg/kg/min, diligent monitoring is indispensable to preempt adverse events and safeguard patient well-being [32].

Individualization of Therapy

Individualizing therapy, particularly in addressing trauma-induced coagulopathy, revolves around tailoring treatment to meet the specific requirements of each patient through rapid diagnostic testing and personalized drug therapy. This approach referred to as goal-directed coagulation therapy, leverages viscoelastic tests such as rotational thromboelastometry (ROTEM) or thrombelastography (TEG), to promptly assess the patient's coagulation status and inform treatment decisions accordingly [33]. By customizing therapy to align with individual patient needs, this personalized approach aims to optimize treatment efficacy, minimize adverse effects, and improve patient outcomes by mitigating risks associated with under-transfusion and over-transfusion [33]. The adoption of early, individualized, and goal-directed therapy, as exemplified in the AUVA Trauma Hospital algorithm, underscores the significance of tailoring coagulation management to suit the unique requirements of each trauma patient, thus enhancing the effectiveness and safety of treatment [33].

Adverse effects and complications

Hypotension and Bradycardia

Hypotension and bradycardia stand out as prominent adverse effects associated with the utilization of esmolol, a selective beta-blocker deployed across various clinical scenarios, including PCC surgery [13,15]. Hypotension, characterized by low blood pressure, can manifest at any esmolol dosage but typically exhibits a dose-dependent relationship [13]. Patients with compromised hemodynamics or those concurrently taking interacting medications face heightened susceptibility. Severe reactions may encompass loss of consciousness, cardiac failure, and even mortality. Maintenance doses surpassing 200 mcg/kg/min are discouraged to regulate ventricular heart rate. Closely monitoring patients, especially those with pre-existing low blood pressure, is imperative. If an unacceptable drop in blood pressure occurs, dosage reduction or cessation of infusion is warranted. Typically, hypotension can be reversed within 30 minutes following dose adjustment or infusion termination [13]. Additionally, bradycardia, inclusive of sinus pause, heart block, severe bradycardia, and cardiac arrest, has been documented with esmolol administration [13,15]. Patients with pre-existing first-degree atrioventricular block, sinus node dysfunction, or conduction disorders may be predisposed to heightened risk. In severe bradycardia, dosage reduction or cessation of infusion is recommended [13]. In a comparative study investigating the effects of esmolol and dexmedetomidine on inducing hypotension and bradycardia during minimally invasive direct coronary artery bypass (MIDCAB) surgery, it was observed that the concurrent administration of neostigmine during esmolol infusion yielded more dependable induction of hypotension and bradycardia compared to esmolol infusion alone [34].

Masking of Hypoglycemia

The concept of masking hypoglycemia pertains to situations where the signs of low blood sugar are not readily apparent, posing potential hazards as individuals may not recognize the condition until it progresses to a severe level. This phenomenon is particularly worrisome in individuals with diabetes, where hypoglycemia unawareness can develop, making it difficult to promptly detect and address low blood glucose levels [35,36]. Masked hypoglycemia poses a significant risk as it can escalate to severe complications such as seizures and coma without timely intervention [35]. Factors contributing to hypoglycemia unawareness include prolonged exposure to low blood glucose levels, previous episodes of hypoglycemia, and impaired functioning of counter-regulatory hormones. Therefore, it is vital for individuals with diabetes to closely monitor their blood glucose levels and seek medical attention if they experience recurring episodes of low blood sugar [36]. Furthermore, certain medications, such as beta-blockers, have the potential to mask the symptoms of hypoglycemia, complicating the identification and management of low blood glucose levels. This highlights the importance of monitoring and awareness of this potential side effect when using such medications [37].

Future directions and research opportunities

Novel Approaches to Hemodynamic Management

Recent years have witnessed the emergence of novel approaches to hemodynamic management in critically ill patients, focusing on continuous and non-invasive monitoring solutions to enhance perioperative care through real-time assessment of cardiovascular function and tissue perfusion [38]. One promising innovation involves the utilization of closed-loop (CL) algorithms for resuscitation in cases of hemorrhagic shock. A 2021 study published in the Journal of Clinical Monitoring and Computing explored CL resuscitation in an experimental animal model and found that while CL treatment arms, delivering fluids and norepinephrine, did not yield statistically significant differences compared to manual clinician resuscitation, they exhibited optimal performance when continuous blood pressure signals were available [38]. Another notable technique is leveraging impedance cardiography (ICG) to manage hypertension. A study conducted in 2022 demonstrated that an algorithm utilizing ICG-derived hemodynamic data was associated with enhanced control of brachial and central blood pressure, along with normalization of cardiovascular hemodynamics in patients with hypertension [39]. Moreover, a novel approach has been developed to establish hemodynamic monitoring in patients receiving support from extracorporeal life support systems (ECLS) for cardiopulmonary failure. This enables continuous assessment of cardiovascular function in these critically ill individuals [40]. In addition to these advancements, other innovative tools for hemodynamic monitoring in shock include pulse contour analysis, esophageal Doppler, and bioreactance. These techniques offer non-invasive or minimally invasive alternatives to conventional invasive monitoring methods [41].

Personalized Medicine in PCC Surgery

Personalized management is pivotal in optimizing outcomes for patients undergoing surgery for PCC and paraganglioma (PGL), ensuring tailored approaches for each individual. Surgery is the primary treatment for localized or primary disease, with the choice of surgical technique influenced by the patient's genotype [42,43]. Notably, individuals with SDHx mutations may necessitate open surgery due to their heightened malignant potential. In contrast, those harboring VHL, RET, or NF1 mutations may benefit from cortical-sparing adrenal surgery to circumvent lifelong glucocorticoid replacement [42,44]. Genetic profiling has facilitated the identification of three primary molecular clusters in PCC/PGL, each characterized by distinct clinical, biochemical, and imaging features [43]. Tailoring management strategies to specific clusters is imperative for prognostic assessment, guiding diagnostic modalities, and delivering personalized treatment regimens and follow-up protocols [43]. In instances where surgery is not viable or in the presence of metastatic disease, secondary treatment options come into play, including chemotherapy with agents like temozolomide or cyclophosphamide + vincristine + dacarbazine [42]. Additionally, radionuclide therapies such as 131I-MIBG, 90Y, and 177Lu-DOTATATE may be considered following an assessment of potential efficacy using 68Ga-DOTATATE PET/CT imaging [42]. Future research endeavors should implement informed, genetically-driven treatment decisions based on germline and somatic mutation testing, targeted drug testing in patient-derived primary cultures, metabolomics, proteomics, and machine learning methodologies. Collaboration between endocrinologists and oncologists equipped with expertise in genomics and genetics is imperative for successfully integrating precision medicine into PCC/PGL management [42].

## Conclusions

In conclusion, managing hemodynamic instability during PCC surgery presents formidable challenges, necessitating effective strategies to mitigate perioperative risks and optimize patient outcomes. This review has underscored the critical role of esmolol, a short-acting beta-blocker, in achieving hemodynamic stability by attenuating the cardiovascular responses to catecholamine release. Through a comprehensive analysis of its pharmacology, mechanisms of action, clinical evidence, and comparative efficacy with other hemodynamic agents, we have highlighted the potential of esmolol to enhance perioperative care in this high-risk patient population. Integrating esmolol into multidisciplinary perioperative protocols, along with meticulous patient selection, individualized dosing, and vigilant monitoring, holds promise for improving surgical outcomes and reducing complications. However, further research is needed to refine dosing regimens, elucidate optimal timing of administration, and explore novel approaches to hemodynamic management in PCC surgery. By fostering collaboration among clinicians, researchers, and industry partners, we can advance our understanding and refine our strategies to meet the evolving needs of patients undergoing surgery for PCC, ultimately enhancing their quality of care and clinical outcomes.
